# Autistic individuals show less grouping-induced bias in numerosity judgments

**DOI:** 10.3389/frcha.2023.1202032

**Published:** 2023-08-30

**Authors:** Antonella Pomè, Themis Karaminis, David C. Burr

**Affiliations:** ^1^ Wahrnehmungspsychologie, Institute for Experimental Psychology, Heinrich Heine University Düsseldorf, Düsseldorf, Germany; ^2^ Department of Psychology, Edge Hill University, Ormskirk, United Kingdom; ^3^ Department of Neuroscience, Psychology, Pharmacology and Child Health, University of Florence, Firenze, Italy

**Keywords:** grouping, connectedness, ASD, numerosity, AQ

## Abstract

**Introduction:**

When items are connected together, they tend to be perceived as an integrated whole rather than as individual dots, causing a strong underestimation of the numerosity of the ensemble. Previous evidence on grouping-induced biases of numerosity has shown a dependency on autistic-like personality traits in neurotypical adults, with a weaker tendency for grouping into meaningful segmented objects in individuals with strong autistic traits. Here we asked whether this result would generalize to the autistic population.

**Methods:**

Twenty-two adults with a diagnosis of Autism Spectrum Disorder (ASD) and 22 matched neurotypical controls judged the numerosity of clouds of dot-pairs connected by thin lines.

**Results:**

Results showed no significant group difference in discrimination precision, suggesting that both groups were equally capable performing the task. However, while connecting pairs of dots at moderate numerosities caused large changes in apparent numerosity in the neurotypical controls, particularly those with low autistic-like traits, it had little effect in the group of autistic participants, suggesting significant differences in numerosity estimation between autistic and neurotypical perception. Consistent with earlier studies, the magnitude of the effect covaried strongly with AQ-defined autistic traits in the neurotypical range, reinforcing the idea that autistic traits predict the strength of grouping.

**Discussion:**

These results provide strong support for the theories of autistic perception that highlight dissimilarities in global vs. local processing, and open the door to study grouping mechanisms indirectly, by asking participants to report on the apparent numerosity rather than on the grouping organization *per se*.

## Introduction

There has been much interest over the past years in using visual illusions to explore atypicalities in sensory processing across multiple domains. Visual illusions exploit unique arrangements of stimulus features that create erroneous perception, which can help clarify the processes by which the brain combines stimulus properties to form a coherent visual percept, often termed a *gestalt*. A particularly valuable visual illusion, used to specify perceptual mechanisms associated with quantity estimation, is the *connectedness* illusion. Items, such as circles or squares, grouped together by thin lines appear to be less numerous (1–5). The illusion has been demonstrated to be effective when connecting the pairs with illusory rather than physical lines (6, 7).

This suggests that numerosity operates on segmented objects defined by global grouping properties, rather than on individual local elements. The effect of grouping is significantly reduced when the items are densely packed (3), suggesting that the effect is restricted to the numerosity range of segregable items. It also affects fMRI responses to numbers (8), adaptation to numbers (9), pupillometry (10), and the dependencies on attentional processes (4). The illusion has been widely explored in neurotypical adults, with both psychophysical and neurophysiological techniques, and recently has been demonstrated that the susceptibility to the illusion extends to non-human animal species (11).

The connectedness illusion has the advantage of tapping grouping mechanisms indirectly, without requiring participants to be aware of and report directly the perceptual organization. This is of critical importance, especially for children and clinical populations, where an indirect measurement may help bypass various cognitive biases or ambiguities in instructions. Judgement of numerosity, on the other hand, is very intuitive and spontaneous (12), making them ideal for clinical testing. Furthermore, there is much evidence that numerosity perception is spontaneous, triggering express saccades (13) and eliciting spontaneous pupillary responses (10).

We have recently demonstrated that the magnitude of the connectedness effect varies with the perceptual style of participants: those scoring high on the self-reported Autism Spectrum Quotient questionnaire (AQ) showed a reduced illusory effect compared with participants with lower autistic traits. This is in line with theories that have linked autism to increased propensity to focus on local object features over global context (5).

Encouraged by our previous finding, we test here the hypothesis that individuals on the Autism Spectrum, as well as neurotypical adults with higher autistic symptom severity, will be less prone to automatically group the connected dots into a single item to reduce overall numerosity. This result is predicted by several theories of visual processing in ASD, including theories that highlight a preference for local stimulus features (14, 15), weakened processing of *central coherence* (16, 17), or reduced weight of prior experiences to the processing of incoming sensory data (“hypo-priors”) (18).

After replicating our previous results (5) on a large group of neurotypical adults with various degrees of autistic traits as measure with the Autism Spectrum Quotient questionnaire (experiment 1), we measured numerosity discrimination in response to the connectedness illusion for two groups of adults: one group of autistic people and one of neurotypical participants matched by age and IQ (experiment 2). The results show that the group with autism (like the neurotypical adults with high autistic traits) had less susceptibility to the illusion, resulting in a less underestimation of numerosity. Our observation supports the hypothesis of weaker propensity for active grouping strategies into meaningful segmented objects in autism.

## Methods

### Participants

In Experiment 1, 43 neurotypical adults [39 females; age (mean ± SD): 19.12 ± 2.29], who reported that they did not have a diagnosis of any neurodevelopmental condition, took part. These participants were first-year undergraduate psychology students, who completed the study for credit.

These participants were assessed on their autistic symptomatology based on the Autism Spectrum Quotient (19), a self-report questionnaire consisting of 50 questions which prompt participants to read a statement and selected the degree to which the statement best described them: “strongly agree,” “slightly agree,” “slightly disagree,” and “strongly disagree”. We used the standard dichotomous scoring algorithm, which adds 1 point to a Total score when a participant's response is suggestive of an autism trait (slightly or strongly agree/disagree depending on positive or negative framing) and 0 points otherwise. Total scores ranged between 6 and 48, with higher scores indicating higher degrees of autistic traits. Almost all participants scored below 32, the threshold above which a clinical assessment is recommended (20). Three were above this threshold, but they did not report an autism diagnosis. AQ scores had a median of 18 (with lower and upper quartiles of 14 and 22.5). The median was used to divide participants into a low- and a high- AQ score group. The demographic characteristics of the two groups are shown in Table 1.

**Table 1:** Mean (standard deviations) in each group of participants; the last column gives the comparison between the two groups of neurotypical adults with various degrees of autistic traits.

	High AQ	Low AQ	Statistical comparison
*N*	25	18	
Gender			*X*^2 ^= 0.74, *p* = 0.38
*n* females: *n* males	23:1^a^	16:2	
Age in years			t(41) = 1.35, *p* = 0.18, logBF = −0.4
Mean (SD)	19.66 (3.21)	18.72 (1.20)	
Range	18–31	18–23	
AQ^b^			t(41) = −5.82, *p* < 0.001, logBF >2,140
Mean (SD)	41.30 (4.38)	16.72 (6.00)	
Range	32–48	6–30	
SRS^c^			t(41) = −3.50, *p* < 0.01, logBF = 26.8
Mean (SD)	119.31 (19.93)	51.13 (22.01)	
Range	73–152	15–88	

^a^ *N* = 1 high AQ gender not specified.

^b^ AQ, Autistic Quotient Questionnaire (20).

^c^ SRS, Social Responsiveness Scale (21).

For experiment 2, we recruited twenty-two autistics from an autism charity research support network (Autistica Discover Network). These were matched on age and gender and cognitive ability (*p* > 0.4) with a group of 22 neurotypical adults recruited from community contacts and selected from an initial larger pool of 27 neurotypical participants. The demographic characteristics of the two groups are shown in Table 2.

**Table 2:** Mean (standard deviations) in each group of participants; the last column gives the comparison between the autistic and non-autistic group of participants.

	ASD	Controls	Statistical comparison
*N*	22	22	
Gender			*x*^2^ = 0.73, *p* = 0.4
*n* females: *n* males	14:6^a^	12:9^a^	
Age in Years			t(42) = 0.62, *p* = 0.54, logBF = −0.7
Mean (SD)	44.55 (14.37)	41.91 (13.72)	
Range	20–62	20–63	
Verbal IQ			t(42) = 3.43, *p* < 0.01, logBF = 1.4
Mean (SD)	62.27 (8.58)	54.04 (7.27)	
Range	47–80	46–80	
Performance IQ			t(42) = −0.72, *p* = 0.47, log BF = −0.7
Mean (SD)	49.09 (6.31)	50.5 (6.09)	
Range	39–66	34–65	
Full-Scale IQ^b^			t(42) = 1.95, *p* = 0.06, log BF = 0.9
Mean (SD)	62.27 (8.58)	54.04 (7.27)	
Range	47–80	46–80	
AQ			t(42) = 15.58, *p* < 0.001, logBF >1,006
Mean (SD)	41.40 (4.38)	16.72 (6.00)	
Range	32–48	6–30	
SRS			t(42) = 10.77, *p* < 0.001, log BF >650
Mean (SD)	119.31 (19.93)	51.13 (22.01)	
Range	73–152	15–88	

^a^ *N* = 2 ASD and *N* = 1 control gender not specified.

^b^ Verbal, Performance and Full-Scale IQ were measured using the Wechsler Abbreviated Scales of Intelligence-2nd edition (22).

All autistic and neurotypical participants in Experiment 2 completed the AQ, as well as Social Responsiveness Scale—Second Edition (21) as a second measure of autistic symptomatology. The SRS-2 asks participants to rate 65 statements relative to their behavior over the past 6 months by ranking the items from 1 “not true” to 4 “almost always true.” The raw scores were calculated and converted to *T*-scores.

All autistic participants scored above the threshold above which a clinical assessment is recommended in both the AQ and the SRS-2 (three participants were excluded from the study for this reason), while all neurotypical participants scored below those thresholds. As expected, in Experiment 2, the autistic group obtained significantly higher AQ (mean (SD) = 41.40 (4.38)) and SRS-2 scores (mean (SD) = 119.31 (19.93)) compared to the non-autistic group (AQ: mean (SD) = 16.72 (6.00), SRS-2: mean (SD) = 51.13 (22.01)), *p* < 0.001.

For participants in experiment 2, cognitive ability was also measured using the Wechsler Abbreviated Scale of Intelligence—Second edition (WASI-II; 22). We obtained a full-scale IQ (FSIQ- 2) measure, based on the Vocabulary task (yielding a verbal comprehension index, VCI), and the Matrix reasoning task (perceptual reasoning index, PRI) raw scores were converted to *T*-scores. An independent-samples *t*-test revealed no significant group differences for FSIQ-2 and PRI, although autistic participants reported a significantly higher performance in VCI compared to controls.

Participants were instructed to complete the session online from a computer in a quiet, distraction-free environment. All participants completed informed consent forms prior to any testing and the experimental procedure was approved by the Science Research Ethics Committee of Edge Hill University (SREC ETH2021–0210). After completion of the informed consent form and the demographic questionnaire, participants were directed to complete the numerosity discrimination tasks through the Pavlovia repository and launch platform (www.pavlovia.org). Instructions were presented in full-screen mode to minimize distractions. Task instructions were delivered to participants as on-screen text. After the experiment was finished, participants were provided with a link to a Qualtrics data collection pipeline that administered the AQ, and the SRS, and at the end they received feedback and compensation (£12 Amazon/John Lewis gift voucher). The WASI-II was administered to participants of experiment 2 separately by the experimenter.

### Stimuli and procedure

**Figure 1:**
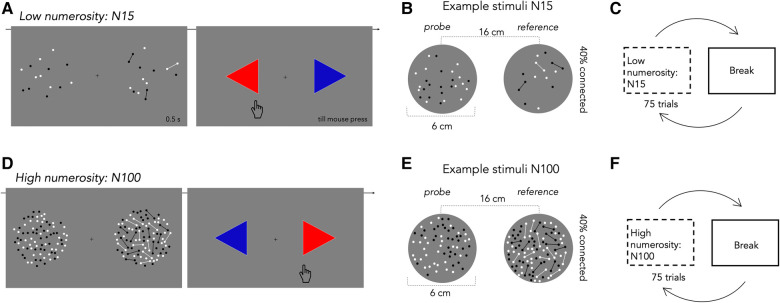
Paradigm and example stimuli used. (**A**) Schematic illustration of one trial in the low numerosity session (N15): two dot clouds were displayed together for 500 ms. After stimulus disappearance, participants indicated which seemed more numerous, pressing the appropriate arrow on the screen. The next trial with the same characteristics then started, after mouse press. (**B**) Example stimuli showing the fixed numerosity reference, with 40% dots connected by thin lines, and the probe varying in numerosity. Each cloud was presented in the periphery at 8 deg of eccentricity from the central fixation cross. (**C**) Participants completed 2 sessions of 75 trials, separated by breaks. **(D–F)** Same as in (**A–C**) but for high numerosity (N100). The two clouds of dots to be discriminated comprised either isolated dots (probe, varying in numerosity) and a fixed-numerosity reference of 100 elements. As before, in total 150 trials were tested.

The task was designed in PsychoPy and administered online via Pavlovia.com. Grouping-induced changes to apparent numerosity were measured by two alternative forced choice (2AFC) numerosity discrimination. Participants were presented with 2 clouds of dots asked to report by mouse press just after stimulus disappearance which of the two stimuli appeared to be more numerous, guessing when uncertain (see Figures 1A,D). Stimuli were presented simultaneously at 8° eccentricity left and right of a fixation cross for 500 ms, too fast for single elements to be serially countable. Stimuli were clouds of small disks of 2.5 mm diameter (subtending 0.25° at 57 cm), half-white, half-black (so luminance did not vary with number, eliminating a potential cue). One of the two dot clouds was the *reference* (randomly left or right), which had fixed numerosity throughout the experimental session; the other was the *probe*, which varied in numerosity, guided by a staircase procedure in which the numerosity of the probe was reduced or increased according to the participant's performance. The reference numerosity could show either moderate (N15) or large (N100) numerosities, depending on the session.

To study grouping mechanisms, 40% of dots in the reference cloud were *connected* to neighboring dots to create dumbbell-like shapes (reference cloud, see Figures 1B,E). For patches containing isolated dots (probe cloud, Figures 1B,E), dot positions were generated on-line to respect the sole condition that two items could not be closer than 2.5 mm (0.25°), preventing dot overlap. For the connected patterns, dot position was calculated in two stages: first couples of dots (40% of the total dots of the reference stimulus) were cast and connected via a line of the same color, with the constraints that line length was between 10 and 15 mm, with no lines crossing; in the second stage, the remaining 60% of dots were cast with the constraint of not overlapping either the other dots or the connecting lines. The connector line width was 0.5 mm.

Participants completed 2 blocks of 75 trials (see Figures 1C,F) in each session (moderate numerosity N15 and large numerosity N100). The 2 sessions were separated by 4 breaks (2 in each session). The experiment could be resumed by button press. On average, the experiment took 40 min.

### Data analysis

Data were analyzed separately for each participant. For each reference numerosity (N15 and N100), the responses were plotted as function of the probe numerosity and fit with a cumulative Gaussian distribution, whose median defines the PSE and the difference in numerosity between 50% and 75% correct responses defines the JND, a measure of precision. The JND divided by the perceived numerosity yields the Weber fraction (WF), a dimensionless index of imprecision that allows comparison of performance across numerosities. The PSE of each participant was used to compute the percent bias for each numerosity:

(1) $$\mathrm{Bias}\;=\;100{\;}^{*}\;(\mathrm{PSE}-N)/N$$

Our main analyses compared data across groups of participants: standard *t*-tests and correlation analyses were complemented with Bayes factor estimation.

Bayes Factors (23) quantify the evidence for or against the null hypothesis as the ratio of the likelihoods for the experimental and the null hypothesis. We express it as the ratio, where negative numbers indicate that the null hypothesis is likely to be true, positive that it is more likely false. By convention, absolute Bayes factors >3 are considered substantial evidence for either the alternate or null hypothesis, >10 strong evidence, and >100 decisive.

## Results

### Experiment 1: grouping strategies in neurotypical adults

**Figure 2:**
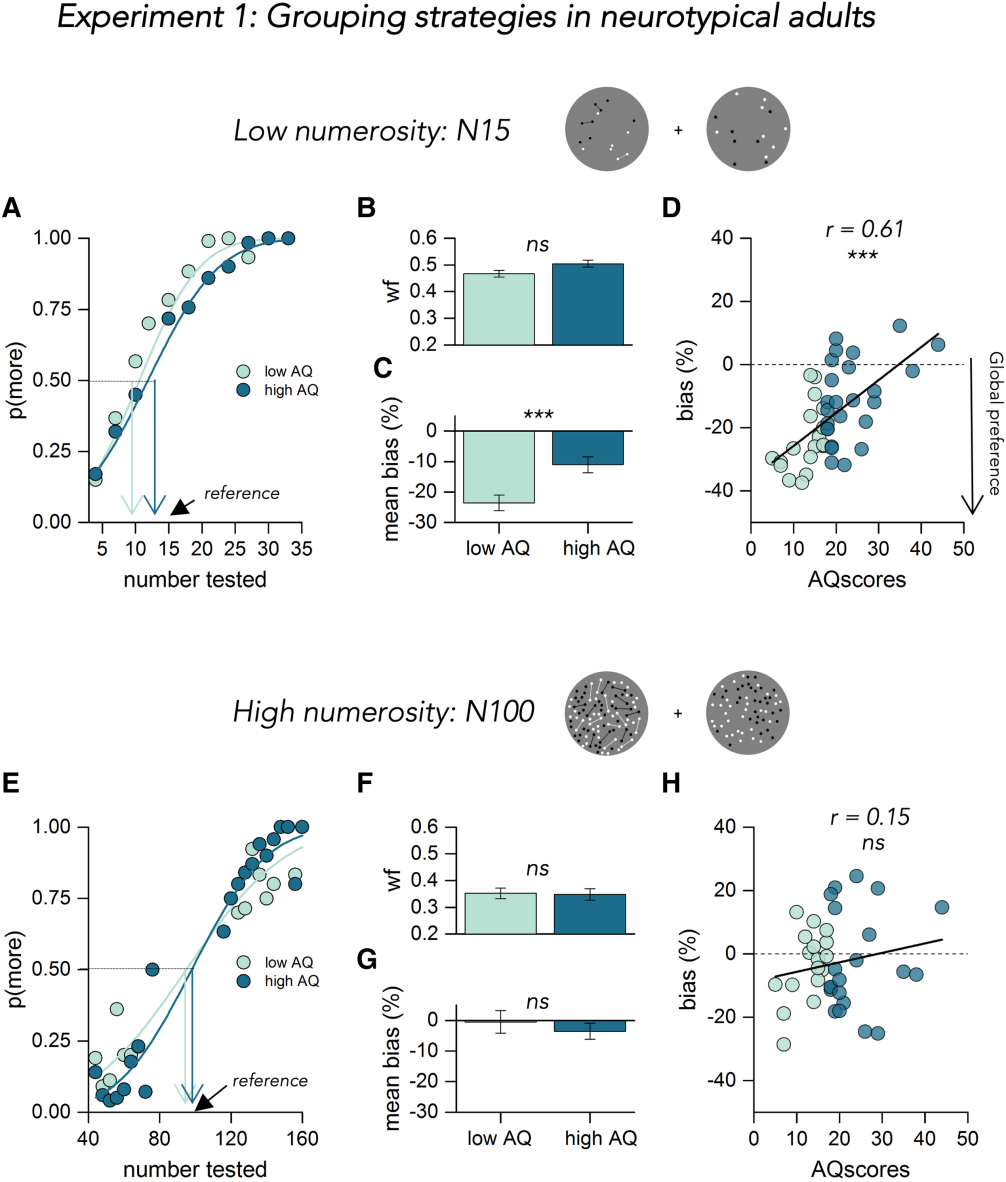
Results of experiment 1. (**A**) Psychometric functions for 2 example participants with low (light blue) and high (dark blue) autistic traits at low numerosity (N15). The functions plot the proportion of trials where the probe appeared more numerous than the reference, as function of probe numerosity (shown in the abscissa). The vertical colored lines show the estimates of the PSE, given by the median of the fitted cumulative Gaussian functions. (**B**) Mean Weber fraction for discriminating numerosity for low (light blue) and high (dark blue) AQ. Error bars = ±1 SEM. Significance values refer to two-sample *T*-test (ns *p* > 0.5). (**C**) Mean response bias (expressed as percentage difference from the reference number) color coded as in (**B**) significance values refer to two-sample *T*-test (***p < 0.001). (**D**) bias, in percentage, at low numerosity plotted against AQ for all participants, color coded according to the median split of their scores. Text insets report Pearson's r and associated *p*-value. Thick black lines show the linear fit through the data. (**E**) Psychometric functions for two example participants with low (light blue) and high (dark blue) autistic traits at high numerosity (N100). Same convention as in (**A**) (**F–H**) same convention as in (**B–D**) but for data of the high numerosity session tested.

We asked participants to discriminate which of two simultaneously presented dot patterns — reference and probe — appeared to be more numerous. The variable probe was always a pattern of isolated dots, while the constant reference comprised dots connected by thin lines, which produces a strong numerosity illusion. Figure 2A shows example psychometric functions for moderate numerosity (N15), for two participants with low (light blue) and high (dark blue) AQ. The functions plot the proportion of trials where the probe appeared more numerous than the reference (N15) as a function of probe numerosity tested. As expected, connecting 40% of dots led to a robust underestimation of the probe numerosity, but only for the low AQ group, around 27% less than the physical numerosity, agreeing with previous literature (1, 2, 6, 3). However, the point of subjective equality (PSE, median at 50%) in the high AQ example participant was very near the physical numerosity of the reference (N15), showing less underestimation of the numerosity tested.

From the psychometric functions of each participant, the PSE (bias) and the JND (precision) was extracted. We first made sure that all participants were able to complete the task by checking their precision measures. We found no statistically significant differences in the Weber fraction (JND/perceived numerosity) between our sample of low (light blue) and high (dark blue) autistic traits [t(41) = –1.46, *p* = 0.08, BF10 = 0.4, Figure 2B]. Moreover, very similar results were obtained when we measured precision as Coefficient of Variation (JND/physical numerosity) [t(41) = –1.85, *p* = 0.07, BF10 = 1.18, not shown as a figure].

We then analyzed the susceptibility to the illusion (expressed as a bias index, eqn1) as a function of autistic traits. The underestimation bias decreased with increasing autistic traits, resulting in a strong correlation between biases and AQ (Figure 2D, *r* = 0.61, *p* < 0.001, BF10 = 1,394.8). This result was also confirmed by the statistical significant difference between the two groups identified via the median split [t(41) = –3.40, *p* < 0.001, BF10 = 21, Figure 2C].

The lower panel of Figure 2 shows the results for high numerosity (N100) stimuli. Here, as previously demonstrated (Anobile et al., 2017), the connectedness effect is much reduced. For 100-dot displays the perceived numerosity was for both groups very close to the physical numerosity. Again, both groups had similar precision thresholds (Weber fractions: *p* = 0.45, BF10 = 0.2, Coefficient of Variation: *p* = 0.28, BF10 = 0.2, Figure 2F). Here there was no correlation between the magnitude of bias and AQ (Figure 2H, *r* = 0.15, *p* = 0.365, BF10 = 0.19), and no significant difference between the average bias of the two groups [Figure 2G, t(41) = –0.62, *p* = 0.27, BF10 = 0.2].

This pattern of results essentially replicates previous findings (3, 5), showing that grouping objects with thin lines lead to an underestimation of perceived numerosity at moderate but not high densities. Grouping occurs when the objects are sparse enough to permit segregation (N15), but not when the objects are too crowded to permit segmentation of the scene into single units. Moreover, the observed effects are linked to autistic symptomatology in individual participants: the grouping process is weaker in individuals with strong autistic traits, suggesting that there might be a major difference between autistic and typical numerosity perception.

### Experiment 2: grouping strategies in adults with and without autism

A group of 22 autistic participants and 22 age- and IQ-matched controls took part in experiment 2. The conditions tested were the same as experiment 1: discrimination of a moderate numerosity (N15) and a high numerosity (N00). We hypothesized that the autistic group would be less prone to the numerosity illusion, resulting in a reduced awareness of the global aspects of stimuli.

**Figure 3:**
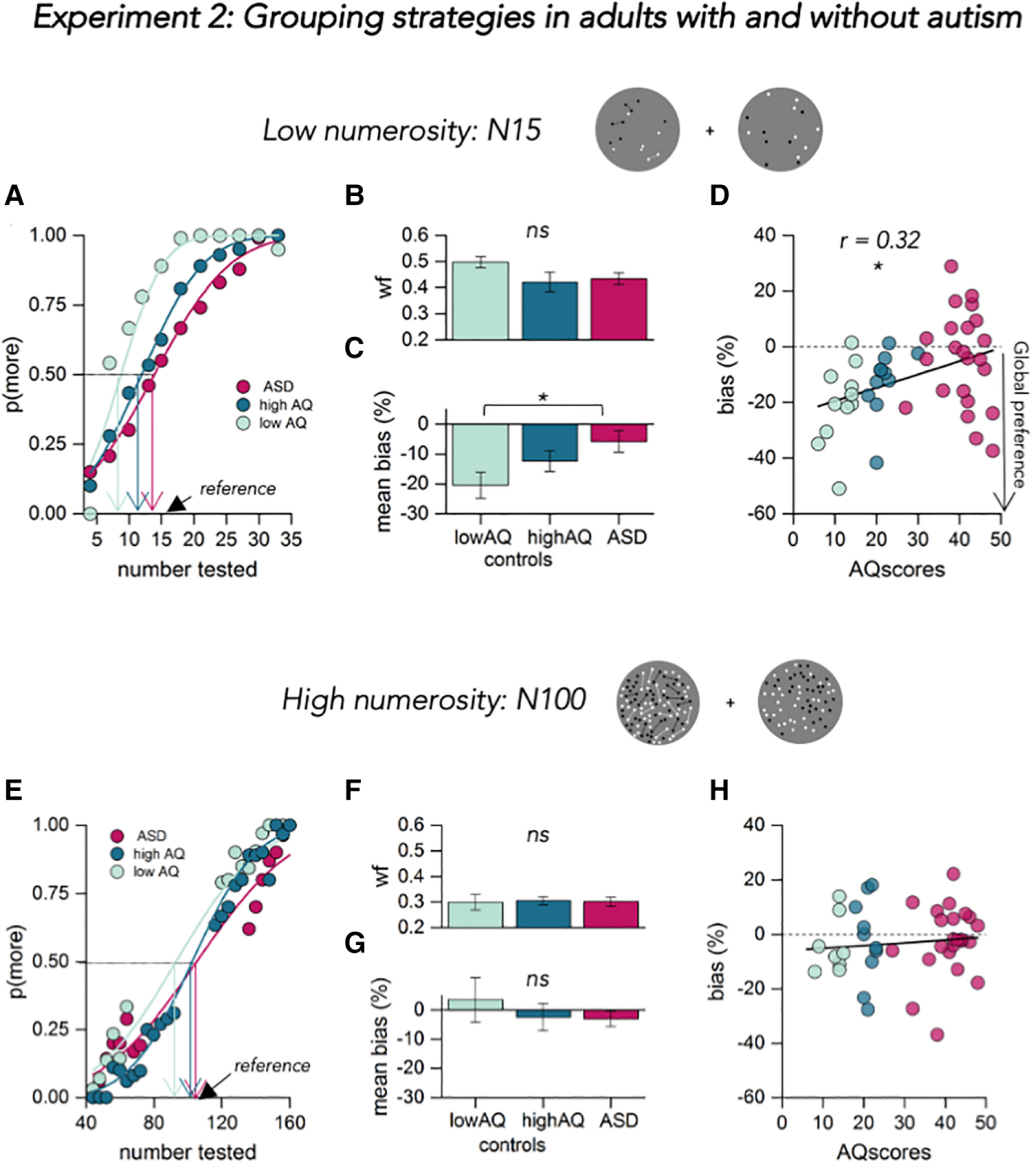
Results of experiment 2. (**A**) Psychometric functions for 3 example participants (one autistic participant in dark pink and one neurotypical with low autistic traits in light blue and one with high autistic traits in dark blue) at low numerosity (N15). The functions plot the proportion of trials where the probe appeared more numerous than the reference, as function of probe numerosity (shown in the abscissa). The vertical colored lines show the estimates of the PSE, given by the median of the fitted cumulative Gaussian functions. (**B**) Mean Weber fraction for discriminating numerosity for ASD (dark pink) and controls (light blue and dark blue respectively for low and high AQ). Error bars = ±1 SEM. Significance values refer to two-sample *T*-test (ns *p* > 0.5). (**C**) Mean response bias (expressed as percentage difference from the reference number) color coded as in (**B**) significance values refer to *post hoc T*-test (*p < 0.05). (**D**) Bias, in percentage, at low numerosity plotted against AQ for all participants, color coded as before. Text insets report Pearson's r values and associated *p*-value. Thick black lines show the linear fit through the data. **(E)** Psychometric functions for three example participants (color coded as in A) at high numerosity (N100). Same convention as in A. (**F–H**) Same convention as in (**B–D**) but for data of the high numerosity session tested.

The procedure was identical to that described for the neurotypical participants of experiment 1. Figure 3A shows psychophysical functions for three example participants: one with autistic diagnosis and two matched-control participants, with low and high autistic traits. For the matched-control participants at moderate numerosity (N15), the PSE changed from 15 to 9, about 40%, for the participant scoring low for autistic traits and from 15 to 11, about 27%, for the participant scoring high. For the example autistic participant, the PSE changed less, from 15 to 14, about 7%. Figure 3C shows the results averaged over all participants. The average bias in the connected patterns at moderate density was about −5% for the autistic group and increased to −12% for high AQ matched controls, and to −21% for low AQ. We found a main effect of group on the underestimation bias [Figure 3C, F(41, 2) = 3.5, *p* = 0.03, BF10 = 1.9]. Participants with low autistic symptoms showed a stronger bias compared to the patients (post-hoc comparison: t = –2.61, *p* = 0.03, BF10 = 3.16). The comparison between ASD patients and all the neurotypical participants was also significant [t(42) = –2.31, *p* < 0.05, BF10 = 1.99].

As with experiment 1, the magnitude of the underestimation bias varied with the autistic quotient of all participants, resulting in a positive relationship between the underestimation effects and AQ (Figure 3D, *r* = 0.32, *p* < 0.05, BF10 = 3.17).

The Weber fraction of the participants, given by the SD of the best-fitting Gaussians to the psychometric functions, normalized by the average perceived quantity, is plotted in Figure 3B. The data show similar discrimination precision between the three groups, with no differences between autistics and controls independently of the autism severity [F(41, 2) = 1.44, *p* = 0.27, BF10 = 2.29], confirming that difference in connectedness effects does not reflect inattention or some other more generic difficulties with judging numerical quantities. This was the same for precision measures expressed as JND/physical numerosity [Coefficient of Variation: F(41, 2) = 1.05, *p* = 0.35, BF10 = 2.95].

Finally, we probed grouping of the dense (N100) dot-patterns (Figure 3 lower panel). Figure 3E shows the performance in this condition for one autistic and one matched control with various degrees of autistic traits. As before, connecting dots with lines at high numerosities lead to weak underestimation of the connected patch: perceived numerosity of the example participants was very close to the physical numerosity of the stimulus (see arrows, Figure 3E). As with experiment 1, we found no differences in mean precision (Figure 3F, WF: F(41, 2) = 0.014, *p* = 0.98, BF10 = 5.75, Coefficient of Variation: F(41,2) = 0.3, *p* = 0.74, BF10 = 4.67) nor in mean underestimation bias [Figure 3G, F(41, 2) = 0.06, *p* = 0.93, BF10 = 5.12] between autistics and controls. Moreover, participant biases at this numerosity did not change as a function of autistic traits measured by the Autism-Spectrum Quotient, as shown in Figure 3H (*r* = –0.04, *p* = 0.823, BF10 = 0.12).

As hypothesized, autistic participants (as well as neurotypical adults with high autistic symptomatology) were less susceptible to the connected numerosity illusion, suggesting that they are less susceptible to grouping effects. This is consistent with their having a more detail-oriented perceptual style (24). Furthermore, all participants showed little grouping at high numerosities, where other perceptual strategies presumably come into play. Indeed, there is considerable evidence that at these higher densities numerosity judgements behave more like texture density discrimination, due to effects similar to visual crowding (25–27).

## Discussion

Uniform *connectedness* has been suggested as a fundamental principle of grouping (28), based on principles such as *proximity*, *similarity*, and *common fate*, first outlined by Gestalt psychologists (29). Specifically, when a region of uniform visual properties is connected, it becomes organized into a single perceptual unit. As here, several studies have shown that connecting dots with lines facilitates grouping, as well as working memory (30). Moreover, spatial regularities and symmetry seem to improve numerosity estimation accuracy (31–33), as well as common color, common faith, and common motion (34, 35).

This study investigated perceptual grouping in autism, using apparent numerosity as an objective measure. The benefit of this technique is to ask participants to report on apparent numerosity of patterns with pairs of adjacent dots connected, rather than on the perceptual organization *per se*, hence tapping grouping mechanisms indirectly.

We first confirmed our previous results by showing that at low-to-moderate numerosities, connecting pairs of dots caused significant alterations in apparent numerosity for neuro-typical individuals with low but not high AQ. However, at higher densities (N100), where the dots are presumably too “crowded” to be efficiently segregated, so other processes come into play, the connected effect was much reduced (3). This reinforces evidence that at higher numerosities, perceptual judgements behave more like texture density discrimination, due to effects similar to visual crowding (25–27).

The second experiment showed that autistic individuals show weak or non-existent connectedness effects, suggesting a major difference in the role of grouping in autistic and neurotypical numerosity perception, supporting theories of weaker grouping mechanisms in ASD. The magnitude of the grouping effects was predicted by autistic symptomatology, in both the neurotypical participants of experiment 1 and the autistic participants of experiment 2. However, all participants in both experiment 1 and 2 showed no connectedness effect when the items in the scene were too many to permit perceptual grouping. This result, together with the fact that we found no differences in discrimination precision (as expressed by the Weber Fractions) in any of the two experiments, is evidence that connected-dot patterns activate different perceptual strategies in autistics and controls that are very unluckily to reflect a general impairment in numerosity judgments or some sort of inattention.

Our results are in line with theories of autistic perception that highlight differences in local rather than global processing (i.e. 36, 37). In accordance with this view, autistic perception systems would be less prone to spontaneously group the connected dots into a single item, reducing overall numerosity. Two possible, not mutually exclusive, explanations could account for weaker susceptibility to this illusion: an emphasis to individual dots, hence ignoring the lines that connect these objects, or a weaker propensity for active grouping strategies into meaningful segmented objects. In a broader sense, our results are also consistent with a less reliance on contextual information by ASD: contextual information contributes to create a percept, including grouping cues which benefit the segmentation of the scene into objects.

Our conclusions, however, seem to be inconsistent with other recent evidence suggesting intact grouping abilities in shape formation in ASD (38, 39). It is not obvious why the two sets of experiments give seemingly conflicting results, especially as both sets of studies tested grouping indirectly. Perhaps the differences are related to the perceptual tasks used to measure the effect: whereas the above studies measured apparent distance, and used a visual search task, our study relied on measurements of perceived numerosity, which seems to be a basic visual attribute that arises spontaneously (40, 12, 13, 10). This may make it more sensitive, and less prone to compensational strategies, to probe implicit grouping mechanisms. Nevertheless, more research is clearly required to unravel fully the different conclusions of these studies.

The current study shows that numerosity judgments are a good method to investigate grouping mechanisms indirectly by asking participants to report the apparent numerosity of a cloud of dots without relying on subjective measures of whether about grouping or perceptual organization. Subjective reports suffer from many problems, such as the difficulty for participants to understand what exactly is required of them, and the difficulty in comparing results between individuals who may use quite different criteria. The concept of numerosity, on the other hand, is one that participants readily understand and spontaneously respond to (12, 13, 10). All groups of participants, both high- and low-AQ neurotypicals as well as those with ASD, performed the task with similar precision, as shown by the Weber fractions. However, there were clear differences in perceived reduction in numerosity for patterns with connected dot-pairs, implicating strong differences in perceptual style measurable with an online numerosity task, particularly in the activation of grouping mechanisms.

To conclude, the current study provides evidence that autism is associated with clear differences in perceptual styles. The reported differences are consistent with the idea that autistic perception is more local than global, in a sense more “veridical”, taking contextual information less into account, as predicted by recent Bayesian accounts (18).

## Data Availability

The raw data supporting the conclusions of this article will be made available by the authors, without undue reservation.
